# Epicatechin Gallate Protects HBMVECs from Ischemia/Reperfusion Injury through Ameliorating Apoptosis and Autophagy and Promoting Neovascularization

**DOI:** 10.1155/2019/7824684

**Published:** 2019-03-06

**Authors:** Bing Fu, Qinghong Zeng, Zhaoting Zhang, Mingyue Qian, Jiechun Chen, Wanli Dong, Min Li

**Affiliations:** ^1^Department of Neurology, The Second People's Hospital of Lianyungang, Lianyungang, Jiangsu 222023, China; ^2^Department of Neurology, The First Affiliated Hospital of Soochow University, Suzhou, Jiangsu 215006, China

## Abstract

Green tea is one of the most beverages with antioxidants and nutrients. As one of the major components of green tea, (-)-epicatechin gallate (ECG) was evaluated for its antioxidative properties in the present study. Cell proliferation assay, tube formation, cell migration, apoptosis, and autophagy were performed in human brain microvascular endothelial cells (HBMVECs) after oxygen-glucose deprivation/reoxygenation (OGD/R) to investigate potential anti-ischemia/reperfusion injury properties of ECG in vitro. Markers of oxidative stress as ROS, LDH, MDA, and SOD were further assayed in our study. Data indicated that ECG could affect neovascularization and promote cell proliferation, tube formation, and cell migration while inhibiting apoptosis and autophagy through affecting VEGF, Bcl-2, BAX, LC3B, caspase 3, mTOR, and Beclin-1 expression. All the data suggested that ECG may be protective for the brain against ischemia/reperfusion injury by promoting neovascularization, alleviating apoptosis and autophagy, and promoting cell proliferation in HBMVECs of OGD/R.

## 1. Introduction

Ischemic stroke is one of the most causes of mortality and disability worldwide which occurs as a result of an obstruction within a blood vessel supplying blood to the brain. Restoration of blood flow involved in the treatment of stroke may lead to reperfusion injury. Ischemia/reperfusion (I/R) often induces tissue damage. After a period of ischemia or lack of oxygen (anoxia or hypoxia), the absence of oxygen and nutrients from blood during the ischemic period creates a condition in which the restoration of circulation leads to oxidative damage and inflammation and through the induction of oxidative stress along with or rather than restoration of normal function. Reperfusion of ischemic tissues is often associated with microvascular injury, and activated endothelial cells produce more reactive oxygen species following reperfusion which results in a subsequent inflammatory response [1]. During the I/R, reactive oxygen species (ROS) lead to the oxidation of proteins, lipids, and DNA, which induce cell proliferation, apoptosis, and necrosis [2, 3]. Superoxide dismutase (SOD) serves a key antioxidant role in cells considering superoxide as one of the principal reactive oxygen species. Malondialdehyde (MDA) is a marker for oxidative stress which results from lipid peroxidation of polyunsaturated fatty acids. Dysfunction of SOD and MDA aggravate oxidative stress and I/R injury [3–5]. LDH is expressed extensively in body tissues and considered a marker of common injuries and disease as it is released during tissue damage. Autophagy is generally activated during I/R and causes autophagic cell death [6]. It is thought that inhibition of autophagy can reduce neurodegenerative damage after focal cerebral ischemia, which seems that the inhibition of autophagy may be a novel strategy to prevent ischemic brain injury [7–9]. Vascular endothelial growth factors (VEGFs) have been reported to participate in vessel repair [10, 11], angiogenesis [10, 12], postischemic brain [13, 14], and neuroprotection in experimental stroke [15], and VEGF signaling pathways are considered as important potential targets for the acute and chronic treatment of stroke [15].

**Figure 1:**
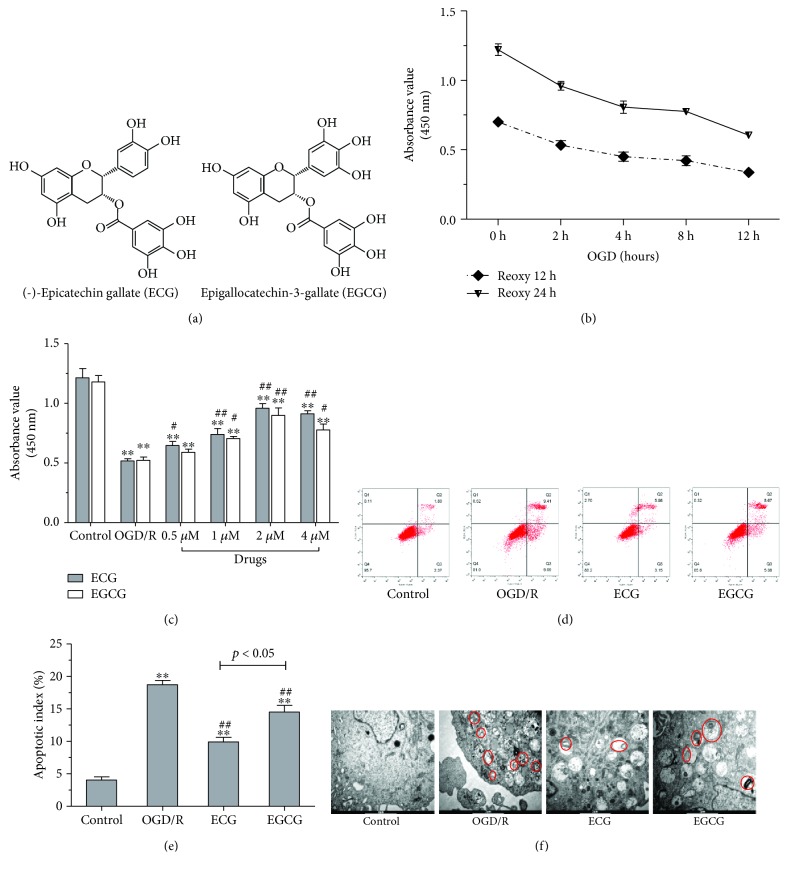
Effect of ECG/EGCG on cell viability, apoptosis, and autophagy in HBMVECs of OGD/R. Chemical structures of ECG and EGCG are shown in (a). Cell viability was assayed by CCK-8 when HBMVECs were cultured in glucose-free culture and oxygen deprivation of 0.5% O_2_ + 5% CO_2_ + 94.5% N_2_ for 2 h, 4 h, 8 h, or 12 h and then reoxygenated for 12 or 24 h in normal medium (b). Cell viability of HBMVECs treated with 0.5, 1, 2, and 4 *μ*M of ECG/EGCG in OGD/R measured by CCK-8 (c). Effect of ECG/EGCG on apoptosis induced by OGD/R, measured by flow cytometry, was explored (d and e). Autophagy was also observed using electron microscopy (f). Data was given as mean ± SD, *n* = 6. ^∗^ *P* < 0.05 and ^∗∗^ *P* < 0.01 compared with control, ^#^ *P* < 0.05 and ^##^ *P* < 0.01 versus cells after OGD/R treatment with solvent (OGD/R or solvent control group).

As reported, green tea consumption may be correlated with a reduced risk of stroke [16, 17]. Most standardized green tea extracts are total polyphenols that contain several antioxidant compounds of polyphenols including (-)-epigallocatechin gallate (EGCG), (-)-epicatechin gallate (ECG), (-)-epigallocatechin (EGC), and (-)-epicatechin (EC) [18]. EGCG and ECG are the above two components of polyphenols extracted from green tea. The chemical structure of ECG is similar to that of EGCG (Figure 1). As previous studies reported, EGCG could scavenge the free radical ions and increase the activity of antioxidant enzymes [19] and reduce upregulation of MMP-9 activity and neuronal damage following transient focal cerebral ischemia induced by middle cerebral artery occlusion (MCAO) in mice [20]. EGCG attenuates oxidative stress responses and promotes autophagy-dependent survival via influencing the balance of the mTOR-AMPK pathway upon endoplasmic reticulum stress [21]. Gundimeda et al. reported that green tea polyphenol precondition resisted cell death induced by oxygen-glucose deprivation (OGD), and the effect of ECG on cell death induced by OGD was consistent with EGCG [22]. ECG exhibited stronger inhibitory activity than did EGCG to oxidation-induced increase in secretory sphingomyelinase involved in many diseases caused by oxidative stress [23]. ECG showed to be more active, playing the important role in the formation of dityrosine (DT) cross-linkages in proteins which is one of the most widely used markers of oxidative stress [24]. In other studies, ECG also showed the maximum antioxidant property [25, 26] among the polyphenols extracted from green tea. On the contrary, the effect of ECG on cerebral I/R injury and its mechanism remain unclear.

In the present study, we first explored the effect of ECG on cell proliferation and apoptosis induced by oxygen-glucose deprivation/reoxygenation (OGD/R) in HBMVECs. Oxidative stress detection, angiogenesis, and cell migration experiments were carried out to evaluate the action of ECG, and the role of ECG in autophagy induced by OGD/R was investigated in our study.

## 2. Materials and Methods

### 2.1. Cells, Reagents, and Antibodies

Human brain microvascular endothelial cells (HBMVECs) were supplied by Angio-Proteomie (MA, USA) and cultured in endothelial cell medium (ECM) (CA, ScienCell) or glucose-free ECM supplemented with endothelial cell growth supplement (ECGS) and 5% fetal bovine serum (FBS). Cells were incubated at 37°C and in an atmosphere of 5% CO_2_ and 95% of air.

(-)-Epicatechin gallate (ECG) (E3893) and epigallocatechin-3-gallate (EGCG) (E4143) were purchased from Sigma (E3893, Sigma-Aldrich, MO, USA). BCA protein assay kit and enhanced chemiluminescence (ECL) were provided by Thermo Scientific (Shanghai, China). Antibodies of GAPDH, VEGF, Bcl-2, LC3B, and FITC goat anti-rabbit antibody used for apoptosis assay were supplied by Abcam (UK). Antibodies of BAX, Caspase 3, mTOR, and Beclin-1 were purchased from Cell Signaling Technology (CST, USA). Secondary antibodies conjugated with horseradish peroxidase were obtained from Abbkine Scientific Co. Ltd (CA, USA).

### 2.2. Oxygen-Glucose Deprivation/Reoxygenation (OGD/R) Model and Treatment

To establish the OGD/R model, HBMVECs were incubated in glucose-free culture under hypoxic conditions of oxygen deprivation of 0.5% O_2_ + 5% CO_2_ + 94.5% N_2_ for 2 h, 4 h, 8 h, or 12 h and then reoxygenated for 12 or 24 h in normal medium. 0.5, 1, 2, and 4 *μ*M of ECG/EGCG were added to the cells prior to hypoxia.

### 2.3. Cell Proliferation Assay

HBMVECs were plated in 96-well plates at a density of 0.5 × 10^4^ cells/well and incubated overnight and subsequently in glucose-free culture under hypoxia oxygenation or treated with various concentrations of ECG/EGCG followed by reoxygenation in complete medium. Cell Counting Kit 8 (CCK-8) (Dojindo, Japan) was utilized to detect cell viability of HBMVECs according to the instructions. In short, 20 *μ*L of CCK-8 solution was added to HBMVECs and incubated at 37°C for 2 h; finally, formazan products were quantified by absorbance at 450 nm detected by a microplate reader (Thermo, MA, USA).

### 2.4. Detection of Apoptosis by Flow Cytometry

We measured cell apoptosis by flow cytometry. HBMVECs after OGD/R and treatment were collected and double-stained with Annexin V-FITC and PI. Apoptosis of cells was measured and analyzed by BD FASAria Cell Sorter flow cytometer (Becton Dickinson) with BD Accuri C6 Software (Becton Dickinson).

### 2.5. Observation of Autophagy by Electron Microscopy

After OGD/R and treatment, HBMVECs were fixed using 2.5% glutaraldehyde and then 1% osmium tetroxide (Sigma-Aldrich, USA) for 30 minutes. Dehydration via acetone and embedding via epoxy embedding medium (Sigma-Aldrich, USA) were performed at room temperature. 1 *μ*m ultrathin sections were made and stained by uranyl acetate (Tianfu Chemical Co. Ltd, China). Autophagy was observed by transmission electron microscopy (FEI, Netherlands).

### 2.6. ROS, LDH, MDA, and SOD Assay

After OGD/R and treatment with ECG, HBMVECs were harvested and cell culture medium was collected. Reactive oxygen species (ROS) of cells was detected using ROS Assay Kit purchased from Beyotime Biotechnology (Shanghai, China) according to the kit instructions of the manufacturer. Lactated hydrogenase (LDH) levels of cell culture medium and malondialdehyde (MDA) and superoxide dismutase (SOD) activity of cells were assayed using assay kits purchased from Jiancheng Co. (Nanjing, China) according to the kit instructions of the manufacturer, and data was calculated using standard curves.

### 2.7. Transwell Migration Assay

To measure cell migration ability, transwell migration assay was performed as previously reported [27, 28]. Briefly, 0.5 × 10^4^ HBMVECs suspended in 200 *μ*L of culture medium with/without drugs were seeded in the upper chamber of transwell (BD) followed by OGD/R and then placed in 24-well plates, and the lower chamber was filled with fresh culture medium. After 48 hours of incubation at 37°C, the filters were taken out gently and cells on the upper surface were removed using cotton swabs. The cells on the underside of transwell filters were stained by 1% of crystal violet (Genemed, USA) for 10 minutes, and photographs were taken (Leica DC 100). Migrating cells stained with crystal violet were collected and measured at 570 nm for quantitative assessment per filter.

### 2.8. Tube Formation Assay

We performed tube formation assay to investigate the effect of ECG on HBMVECs. 50 *μ*L of ice-cold matrigel (BD) and serum-free culture medium were mixed and added to 96-well plates. After treatment and OGD/R, HBMVECs with 1 × 10^4^/well in 200 *μ*L of medium were added to the plates and incubated for 5 h, and the tube networks were photographed and quantified by the number of tube formations using ImageJ software [29].

### 2.9. RNA Extraction and Real-Time Quantitative PCR (qRT-PCR)

Total RNA of cells was extracted using RNA isolation reagent (Invitrogen, USA) and then was reverse-transcribed using One Step RT-qPCR Kit (Sangon Biotech, China) according to the manufacturer's protocol. Next, equal amounts of cDNA were used for RT-qPCR. PCR reaction and real-time detection were performed using iQ5 Real-time Quantitative PCR (Bio-Rad, USA). As shown in Table 1, the appropriate forward and reverse real-time PCR primers were used for GAPDH, VEGF, Bcl-2, BAX, LC3B, Caspase 3, mTOR, and Beclin-1. The real-time PCR cycles included predenaturation at 95°C for 5 min, 40 cycles of denaturation at 95°C for 20 sec, annealing at 60°C for 30 sec, and extension at 72°C for 30 sec. 2^−ΔΔCt^ (ΔΔCt = (C_mRNA_–Ct_GAPDH_) − (control − Ct_GAPDH_)) was used to quantify the relative expression of target mRNA.

### 2.10. Western Blotting

After OGD/R and treatment with ECG, HBMVECs were harvested and cell extracts were prepared. Briefly, cells were lysed in lysis buffer (20 mM Tris-pH 7.5, 150 mM NaCl, 1% Triton X-100) followed by centrifugation for 3 min at 10,000 × *g* at 4°C. The supernatant was collected and transferred electrophoretically to a polyvinylidene fluoride membrane (PVDF) (Millipore, Shanghai, China). The membranes were blocked with 5% dry milk and subsequently incubated with primary antibodies against GAPDH (Abcam, UK), VEGF (Abcam, UK), Bcl-2 (Abcam, UK), BAX (CST, USA), Caspase 3 (CST, USA), mTOR (CST, USA), LC3B (Abcam, UK), and Beclin-1 (CST, USA) overnight at 4°C. The membranes were subsequently washed and incubated with secondary antibodies conjugated with horseradish peroxidase. The immunoreactive bands were visualized by enhanced chemiluminescence (Thermo Scientific, Shanghai, China) and analyzed by automatic chemiluminescence image analysis system (Tanon, China). The results were normalized to GAPDH.

### 2.11. Statistical Analysis

All experiments were repeated at least three times, and all data are shown as means ± standard deviations (SD) of six independent samples. The significant difference in two groups was evaluated using the Mann–Whitney test or nonparametric test, and a *P* value of 0.05 or less is considered statistically significant.

## 3. Results

### 3.1. Effect of ECG/EGCG on Cell Viability, Apoptosis, and Autophagy of HBMVECs Undergoing OGD/R

The effect of ECG/EGCG on cell viability, autophagy, and apoptosis in HBMVECs induced by OGD/R was explored in our study. The OGD/R model in HBMVECs was first made, and the effect of ECG/EGCG was evaluated. It is shown in Figure 1(b) that cell viability was decreased time-dependently during OGD/R. Cell viability of HBMVECs induced by 12 h-OGD combined with 12 h reperfusion or 12 h OGD combined with 24 h reperfusion was decreased to 48.1% and 49.6% of control cells individually. We choose OGD/R of 12 h OGD and 12 h reperfusion for the following study. Cell viability of HBMVECs after OGD/R treated with ECG/EGCG showed a dose-dependent increase in doses of 0.5 *μ*M to 4 *μ*M compared with the solvent control group (OGD/R) (Figure 1(c)). It was 0.5 *μ*M ECG not EGCG which showed a significant effect on promoting cell viability compared with the solvent control group (OGD/R) (*P* < 0.05). 2 *μ*M ECG/EGCG showed the most significant promotion of cell viability effect. Cell viability in 4 *μ*M ECG/EGCG-treated cells showed a decreasing trend, and EGCG was more obvious. We chose 2 *μ*M of ECG/EGCG for further apoptosis and autophagy experiments. As shown in Figures 1(d) and 1(e), both ECG and EGCG showed inhibition of OGD/R-induced apoptosis. In ECG-treated cells, the rate of apoptosis was significantly decreased compared with the EGCG-treated group. OGD/R-induced autophagy was observed by transmission electron microscopy, and the effect of ECG on autophagy was similar to that on apoptosis (Figure 1(f)). ECG was chosen for our further study.

### 3.2. Effect of ECG on ROS, LDH, MDA, and SOD of HBMVECs after OGD/R

**Figure 2:**
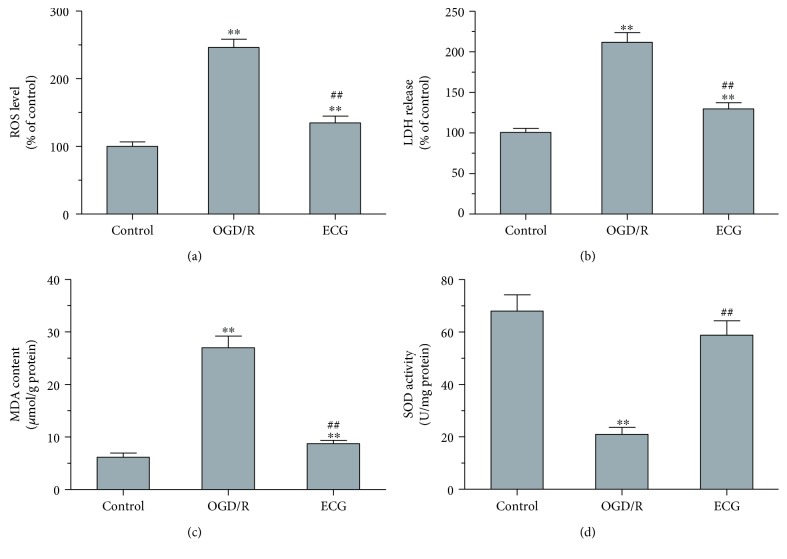
ECG affected ROS, LDH, MDA, and SOD in HBMVECs after OGD/R. Levels of ROS (a) and MDA (c), SOD activity (d) in cells and LHD releasing level (b) were detected using assay kits correspondingly. Data was given as mean ± SD, *n* = 6. ^∗^ *P* < 0.05 and ^∗∗^ *P* < 0.01 compared with control, ^#^ *P* < 0.05 and ^##^ *P* < 0.01 versus cells after OGD/R treatment with solvent (OGD/R or solvent control group).

To evaluate the antioxidant activity of ECG, ROS, LDH, MDA, and SOD assay was performed in our study. OGD/R induced an ROS level about 2.5-fold to control (normal), whereas ECG downregulated ROS significantly compared with the OGD/R group (*P* < 0.01) (Figure 2(a)). OGD/R induced oxidative stress, and the LDH level was used to assess cell death. ECG treatment significantly inhibited LDH leakage induced by OGD (*P* < 0.01) (Figure 2(b)). As shown in Figure 2(c), OGD/R upregulated intracellular MDA levels compared with control which was significantly decreased by ECG treatment (Figure 2(c)). As MDA was considered an indicator of lipid peroxidation, ECG may show inhibition of lipid peroxidation in HBMVECs that underwent OGD/R. In the OGD/R group, the SOD activities were significantly decreased compared with the control group (Figure 2(d)). ECG treatment significantly increased SOD activity (*P* < 0.01).

### 3.3. ECG Prompted Migration and Tube Formation of HBMVECs after OGD/R

**Figure 3:**
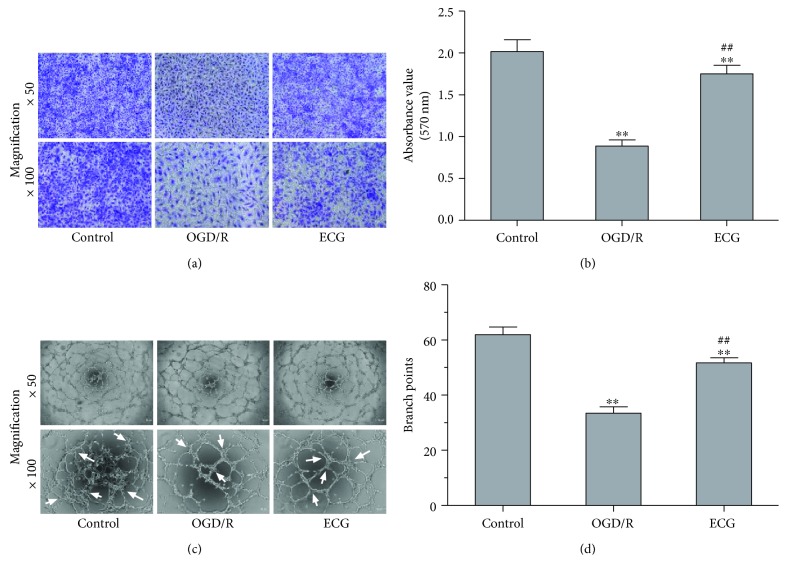
ECG prompted migration and tube formation of HBMVECs after OGD/R. Cell migration of HBMVECs after OGD/R and treated with ECG was assayed using the Transwell method (a), and migrating cells were stained with crystal violet and measured at 570 nm for quantitative analysis (b). Tube formation of HBMVECs after OGD/R and treatment with ECG was performed (c), and tubes were quantified as analysis branch points as indicated by white arrows using ImageJ software (d). Data was given as mean ± SD, *n* = 6. ^∗^ *P* < 0.05 and ^∗∗^ *P* < 0.01 compared with control, ^#^ *P* < 0.05 and ^##^ *P* < 0.01 versus cells after OGD/R treatment with solvent (OGD/R or solvent control group).

To explore ECG on neovascularization in vitro, migration assay and tube formation were performed. OGD/R induced HBMVEC migration ability decreasing significantly (*P* < 0.01), and ECG prompted cell migration significantly (*P* < 0.01) even though it did not reverse OGD/R-induced inhibition of migration (Figures 3(a) and 3(b)). Cell tube formation capacity assayed by branch point number [30] was significantly decreased in the OGD/R group (*P* < 0.01) (Figures 3(c) and 3(d)). The number of tube formation in the ECG-treated group was about 1.55-fold of that in the OGD/R group.

### 3.4. Effect of ECG on mRNA and Protein Expression of VEGF, Bcl-2, BAX, LC3B, Caspase 3, mTOR, and Beclin-1

**Figure 4:**
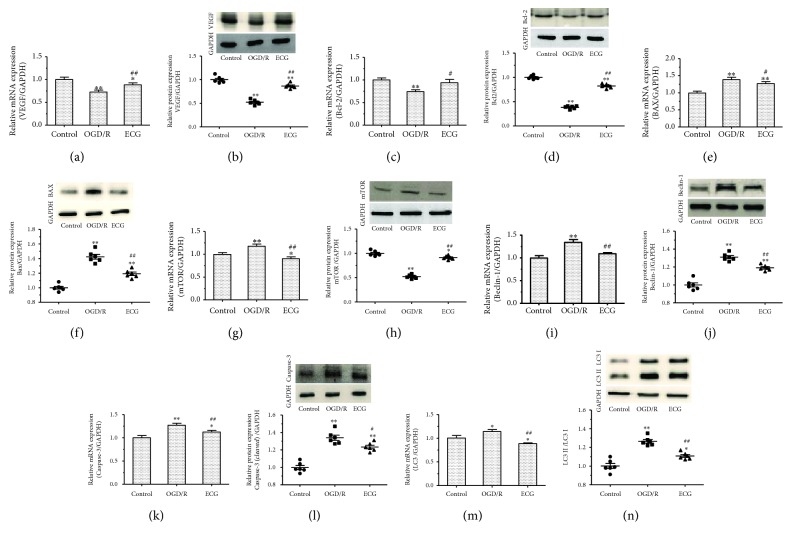
The effect of ECG on mRNA and protein expression of VEGF, Bcl-2, BAX, LC3B, Caspase 3, mTOR, and Beclin-1 in HBMVEs after OGD/R. (a), (c), (e), (g), (i), (k), and (m) represent relative mRNA expression of VEGF, Bcl-2, BAX, LC3B, Caspase 3, mTOR, and Beclin-1 measured by qPCR. Protein expression of VEGF, Bcl-2, BAX, LC3B, Caspase 3, mTOR, and Beclin-1 determined by western blotting is shown in (b), (d), (f), (h), (j), (l), and (n) correspondingly. Data was given as mean ± SD, *n* = 6. ^∗^ *P* < 0.05 and ^∗∗^ *P* < 0.01 compared with control, ^#^ *P* < 0.05 and ^##^ *P* < 0.01 versus cells after OGD/R treatment with solvent (OGD/R or solvent control group).

mRNA and protein expression of vascular endothelial growth factor of VEGF, cell proliferation, and apoptosis associated Bcl-2, BAX, and Caspase 3, as well as LC3B, mTOR, and Beclin-1 which is related to autophagy was measured in our study. Compared with control, mRNA expression of VEGF (Figure 4(a)) and Bcl-2 (Figure 4(c)) decreased significantly (*P* < 0.01), whereas BAX (Figure 4(e)), mTOR (Figure 4(g)), Beclin-1 (Figure 4(i)), Caspase 3 (Figure 4(k)), and LC3B (Figure 4(m)) mRNA expression increased significantly (*P* < 0.01) in the OGD/R group. ECG tends to inhibit downregulation of VEGF and Bcl2 as well as upregulation of BAX, mTOR, Beclin-1, Caspase 3, and LC3B induced by OGD/R.

To investigate the effect of ECG on the expression of OGD/R-related proteins and the mechanism involved, we also measured protein expressions of VEGF, Bcl2, BAX, mTOR, Beclin-1, cleaved Caspase 3, and LC3B. The expression of VEGF (Figure 4(b)), Bcl2 (Figure 4(d)), and mTOR (Figure 4(h)) in the OGD/R group was 0.52-, 0.38-, and 0.51-fold to that in the control group. As for BAX (Figure 4(f)), Beclin-1 (Figure 4(j)), and cleaved Caspase 3 (Figure 4(l)) protein expression, there was a 1.42-, 1.31-, and 1.34-fold expression to the control group, respectively, in OGD/R group. Relative content of LC3II to LC3I was significantly upregulated in the OGD/R group compared with control (*P* < 0.01) (Figure 4(n)). ECG treatment attenuated regulation, up or down, induced by OGD/R (*P* < 0.05 or *P* < 0.01).

## 4. Discussion

Green tea is one of the most beverages with antioxidants and nutrients that have powerful effects on the body which include improving brain function [31–33], fat loss [34], lower risk of cancer [35, 36], and many other impressive benefits [37, 38]. There are many reports that green tea or its extract has protective effects on ischemia-reperfusion injury of the brain [39, 40], heart [41, 42], kidney [43], liver [44, 45], and intestine [46]. Polyphenols exhibit antioxidative and anti-inflammation effects in a lot of studies in vivo and in vitro [47, 48]. Green tea and its extracts are rich in polyphenols. EGCG and ECG are the major two polyphenols included in green tea [49]. The chemical structure of ECG is similar to EGCG (Figure 1(a)). Cerebral microcirculation plays an important role in substance exchange and oxygen supply, and reperfusion of ischemic tissues is often associated with microvascular injury.

In the present study, we first established an OGD/R model in HBMVECs in vitro. Cell viability decreased time-dependently during OGD/R, and therefore, we choose time conditions of OGD/R of 12 h OGD and 12 h reperfusion, under which cell viability decreased to about 50% to control and OGD/R in the shortest time simultaneously. The cell viability of HBMVECs after OGD/R treatment with 0.5 *μ*M to 4 *μ*M of ECG/EGCG showed a dose-dependent increase compared to the solvent control group (*P* < 0.05). 0.5 *μ*M of ECG showed a significant effect on promoting cell viability compared to the solvent control group, and no significant effect of 0.5 *μ*M of EGCG showed. 2 *μ*M ECG/EGCG showed the most significant promoting cell viability effect, which was similar to Gundimeda et al.'s report [22], and 4 *μ*M ECG/EGCG-treated cells tend to show a decreasing cell viability. 2 *μ*M of ECG/EGCG was chosen for the followed apoptosis and autophagy experiments. ECG and EGCG both showed inhibition of OGD/R-induced apoptosis and autophagy. Compared with the solvent control group (OGD/R), the apoptosis rate in the ECG-treated group decreased to its 53.5 percent and 25.2 percent decrease in the EGCG-treated group. The apoptosis rate of EGCG-treated cells was about 1.4-fold higher than that of ECG-treated ones. It is similar to apoptosis; autophagic bodies in ECG- and EGCG-treated cells were significantly less than those in the solvent control group, and EGCG-treated cells produced even more autophagic bodies than did ECG-treated cells. During ischemia/reperfusion injury, oxidative stress may induce epithelial cell apoptosis [50–53] and/or autophagy [51, 54, 55]. Autophagy is a widely existing metabolic and strictly regulated process, and excessive or deficient autophagy may contribute to pathogenesis [56]. Autophagy exerts dual roles in cell death or survival during an ischemic insult or preconditioning during which autophagy is activated [57]. Autophagy may be triggered by preconditioning or lethal ischemia and then interacts with apoptotic and necrotic signaling pathways to regulate cell death. In addition, autophagy may also maintain cell function by removing protein aggregates or damaged mitochondria. Autophagy is also a double-edged sword in angiogenesis. Induction autophagy could inhibit retinal neovascularization in vitro and in vivo to improve oxygen-induced retinopathy [58]. ECG showed a more antagonistic effect on OGD/R-induced apoptosis and autophagy than did EGCG. The effect of EGCG on I/R-induced apoptosis and autophagy was consistent to previous studies [59, 60]. We firstly evaluated the effects of ECG/EGCG on cell viability, apoptosis, and autophagy of HBMVECs after OGD/R. And our results suggested that ECG may show a more significant effect than EGCG. Considering that ECG exhibited the maximum antioxidant property [25, 26] among the polyphenols extracted from green tea, we chose ECG for our following study. The chemical structure of ECG is similar to EGCG; however, it seemed that ECG acts better than EGCG on cerebral ischemia/reperfusion injury in vitro. Ghosh et al. reported that aromatic interactions, hydrophobic interactions, the radical scavenging activity, and autoxidation of polyphenols are likely to be the major reasons for ECG being the most effective inhibitor of fibrillation [26]. To evaluate the scavenging effects of ECG and EGCG, the results of the previous study of Kondo et al. indicated that ECG can be converted to an anthocyanin-like compound after cleavage of the gallate moiety; however, EGCG can be converted to an anthocyanin-like compound followed by cleavage of the gallate moiety by oxidation [25]. Active oxygen including superoxide (O2^−^) would not be produced in EGCG, but can be produced in ECG [25]. The radical scavenging property of green tea polyphenols lies in the order of ECG > EGCG, which accounts for better protective activity of ECG from ischemia/reperfusion injury than EGCG [26]. Data of further studies for ECG showed that ECG has antioxidant activity that upregulation of ROS, LDH, and MDA as well as downregulation of SOD induced by OGD/R were almost reversed by treatment of ECG. Results on the investigation of ECG effect on neovascularization in vitro indicated that ECG could promote HBMVECs undergoing OGD/R migration and tube formation significantly (*P* < 0.01). We further measured mRNA and protein expression of vascular endothelial growth factor of VEGF, cell proliferation, and apoptosis associated Bcl-2, BAX, and Caspase 3, as well as LC3B, mTOR, and Beclin-1 associated with autophagy. It was found that mRNA and protein expression of VEGF and Bcl-2 decreased and BAX, Beclin-1, and Caspase 3 increased significantly after OGD/R; however, ECG treatment alleviated even eliminated the influence of OGD/R on HBMVECs. OGD/R also induced mRNA of LC3B and relative protein expression of LC3II to LC3I increase, all of which was downregulated by ECG. It is worth noting that the tendency of mRNA and protein expression of mTOR change was inconsistent in the OGD/R group (solvent control group) or ECG-treated group. Different regulation mechanisms as synthesis and degradation rates, acting on both the synthesized mRNA and the synthesized protein, affect the amount of the two molecules differentially, and the relation between mRNA and protein maybe not strictly linear, but has a more intrinsic and complex dependence [61, 62]. The OGD/R group induced protein expression of mTOR decrease while ECG antagonized OGD/R induction generally.

ECG is one of the major polyphenolic components of green tea only second to EGCG which was reported to have protective effects on ischemia-reperfusion injury by attracting cell proliferation, apoptosis, and autophagy in vitro and in vivo. It has been shown that a high concentration (more than 10 *μ*M) of EGCG causes a significant decrease in the number of viable cells and induces human embryonic kidney cells (HEK293T) apoptosis and autophagy [21]; however, low concentrations (2 *μ*M) of ECG and EGCG enhance the viability of PC12 cells undergoing OGD/R [22]. In another study, 2 *μ*M of EGCG and ECG showed an effect against radical oxidation in aqueous media [25]. Therefore, the doses of 0.5, 1, 2, and 4 *μ*M of ECG/EGCG for treatment were involved in our study. Our results suggested that ECG tends to play a more important role than does EGCG in OGD/R-induced HBMVECs in cell proliferation, apoptosis, and autophagy. In our study, we have also investigated the effect of ECG on neovascularization. Our results indicated that ECG could promote HBMVEC migration and tube formation in the OGD/R-induced group, and VEGF expression was upregulated in the ECG-treated OGD/R group compared with solvent control. Although ECG showed an effect on I/R injury in vitro in our study, we would evaluate the role of ECG in vivo against I/R injury in our further study. Considering that the content of ECG is less than that of EGCG and treatment of a lower dose of 0.2 *μ*M combined with other polyphenolic components of green tea showed about equivalent effects on protection cells from I/R injury-induced cell death, which was mentioned in the previous study [22], exploring the effect of ECG combined with EGCG on I/R injury in vitro and in vivo will be involved in our further study too.

In conclusion, the results from the present study indicated that ECG could promote cell proliferation, intervene apoptosis and autophagy, and promote cell migration & tube formation and expression of VEGF to affect neovascularization in protection HBMVECs from I/R injury. ECG also showed oxidation resistance through affecting OGD/R-induced ROS, LDH, MDA, and SOD change and antagonized OGD/R induction to mRNA and protein expression of cell proliferation and apoptosis associated Bcl-2, BAX, and Caspase 3, as well as LC3B, mTOR, and Beclin-1 related to autophagy. ECG may protect HBMVECs from OGD/R and offer a promising therapeutic approach for the treatment of I/R injury.

## Figures and Tables

**Table 1:** Primers sequences, size of the amplification product, and NCBI reference sequence.

Gene	Sequence		AMPL. size	NCBI ref. seq.
GAPDH	Fw:	5′- GGAGCGAGATCCCTCCAAAAT -3′	197 bp	NM_001256799
	Rev:	5′- GGCTGTTGTCATACTTCTCATGG -3′		
VEGF	Fw:	5′- AGGGCAGAATCATCACGAAGT -3′	75 bp	NM_001171627
	Rev:	5′- AGGGTCTCGATTGGATGGCA -3′		
Bcl-2	Fw:	5′- GGTGGGGTCATGTGTGTGG-3′	89 bp	NM_000657
	Rev:	5′- CGGTTCAGGTACTCAGTCATCC-3′		
BAX	Fw:	5′- CCCGAGAGGTCTTTTTCCGAG-3′	155 bp	NM_138763
	Rev:	5′- CCAGCCCATGATGGTTCTGAT-3′		
LC3B	Fw:	5′- GATGTCCGACTTATTCGAGAGC-3′	167 bp	NM_022818
	Rev:	5′- TTGAGCTGTAAGCGCCTTCTA-3′		
Caspase 3	Fw:	5′- CATGGAAGCGAATCAATGGACT-3′	139 bp	NM_004346
	Rev:	5′- CTGTACCAGACCGAGATGTCA-3′		
mTOR	Fw:	5′- ATGCTTGGAACCGGACCTG-3′	173 bp	NM_004958
	Rev:	5′- TCTTGACTCATCTCTCGGAGTT-3′		
Beclin-1	Fw:	5′- CCATGCAGGTGAGCTTCGT-3′	215 bp	NM_003766
	Rev:	5′- GAATCTGCGAGAGACACCATC-3′		

Fw = forward; Rev = reverse.

## Data Availability

All the tables and figures used to support the findings of this study are included within the article.

## Abbreviations

ECG: (-)-Epicatechin gallate
EGCG: Epigallocatechin-3-gallate
OGD/R: Oxygen-glucose deprivation/reoxygenation
HBMVECs: Human brain microvascular endothelial cells
LDH: Lactated hydrogenase
ROS: Reactive oxygen species
MDA: Malondialdehyde
SOD: Superoxide dismutase
I/R: Ischemia/reperfusion
VEGF: Vascular endothelial growth factor
mTOR: Mammalian target of rapamycin.

## Supplementary Materials

Supplementary Materials Western blotting after OGD/R and treatment with ECG. HBMVECs were harvested, and cell extracts were prepared. Briefly, cells were lysed in lysis buffer (20 mM Tris-pH 7.5, 150 mM NaCl, and 1% Triton X-100) followed by centrifugation for 3 min at 10,000 × *g* at 4°C. The supernatant was collected and transferred electrophoretically to a polyvinylidene fluoride membrane (PVDF) (Millipore, Shanghai, China). The membranes were blocked by 5% dry milk and subsequently incubated with primary antibodies against GAPDH (Abcam, UK), VEGF (Abcam, UK), Bcl-2 (Abcam, UK), BAX (CST, USA), Caspase 3 (CST, USA), mTOR (CST, USA), LC3B (Abcam, UK), and Beclin-1 (CST, USA) overnight at 4°C. The membranes were subsequently washed and incubated with secondary antibodies conjugated with horseradish peroxidase. The immunoreactive bands were visualized by enhanced chemiluminescence (Thermo Scientific, Shanghai, China) and analyzed by automatic chemiluminescence image analysis system (Tanon, China). The results were normalized to GAPDH. Results on the effect of ECG on mRNA and protein expression of VEGF, Bcl-2, BAX, LC3B, Caspase 3, mTOR, and Beclin-1 mRNA and protein expression of vascular endothelial growth factor of VEGF, cell proliferation, and apoptosis associated Bcl-2, BAX, and Caspase 3, as well as LC3B, mTOR, and Beclin-1 which is related to autophagy were measured in our study. Compared with control, mRNA expression of VEGF (Figure 4(a)) and Bcl-2 (Figure 4(c)) decreased significantly (*P* < 0.01), whereas BAX (Figure 4(e)), mTOR (Figure 4(g)), Beclin-1 (Figure 4(i)), Caspase 3 (Figure 4(k)), and LC3B (Figure 4(m)) mRNA expression increased significantly (*P* < 0.01) in the OGD/R group. ECG tends to inhibit downregulation of VEGF and Bcl2 as well as upregulation of BAX, mTOR, Beclin-1, Caspase 3, and LC3B induced by OGD/R. To investigate the effect of ECG on expression of OGD/R related proteins and the mechanism involved, we also measured protein expression of VEGF, Bcl2, BAX, mTOR, Beclin-1, cleaved Caspase 3, and LC3B. The expression of VEGF (Figure 4(b)), Bcl2 (Figure 4(d)), and mTOR (Figure 4(h)) in the OGD/R group was 0.52-, 0.38-, and 0.51-fold to that in the control group. As for BAX (Figure 4(f)), Beclin-1 (Figure 4(j)), and cleaved Caspase 3 (Figure 4(l)) protein expression, there was a 1.42-, 1.31-, and 1.34-fold expression to the control group, respectively, in the OGD/R group. The relative content of LC3II to LC3I was significantly upregulated in the OGD/R group compared with control (*P* < 0.01) (Figure 4(n)). ECG treatment attenuated up- or downregulation of protein expression induced by OGD/R (*P* < 0.05 or *P* < 0.01).
