# Supplementary figures and images for: Epicatechin Gallate Protects HBMVECs from Ischemia/Reperfusion Injury through Ameliorating Apoptosis and Autophagy and Promoting Neovascularization
Source: Oxid Med Cell Longev. 2019 Mar 6;2019:7824684. doi: 10.1155/2019/7824684 (PMC6431361; doi:10.1155/2019/7824684)

1) VEGF

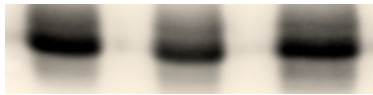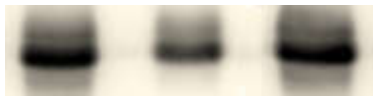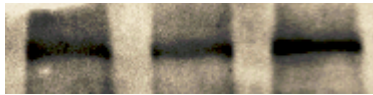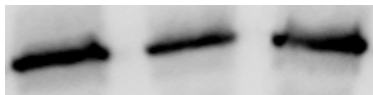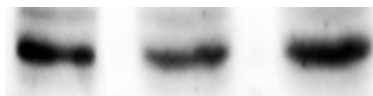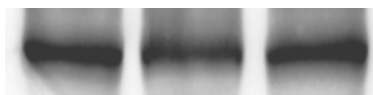

GAPDH

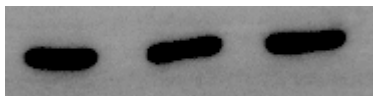

2) Bcl-2

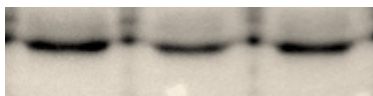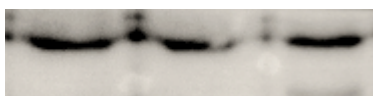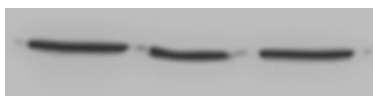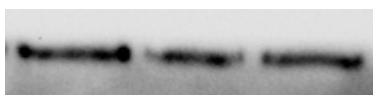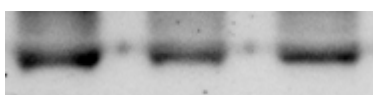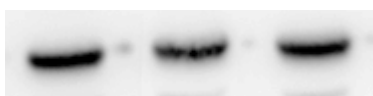

GAPDH

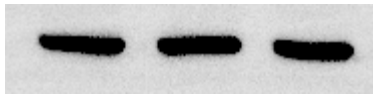

3)BAX

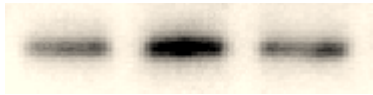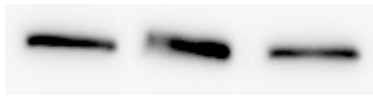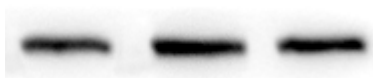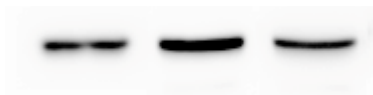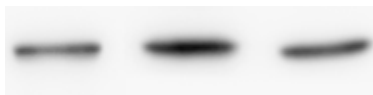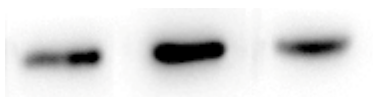

GAPDH

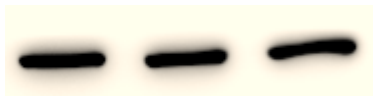

4)mTOR

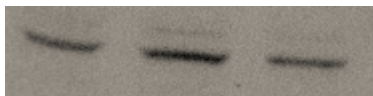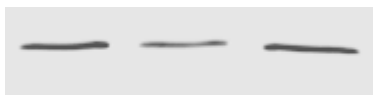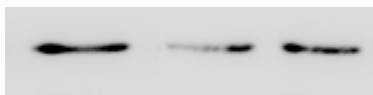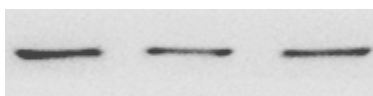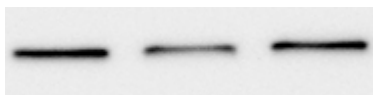

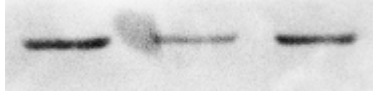

GAPDH

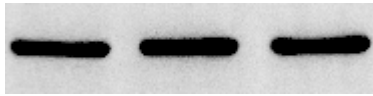

5) BECN-1

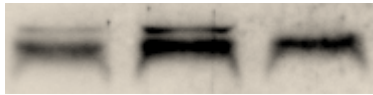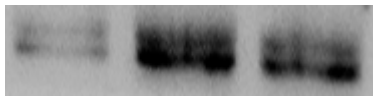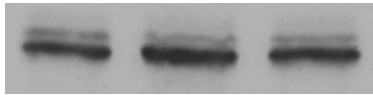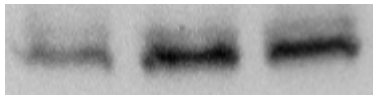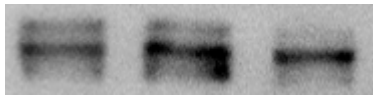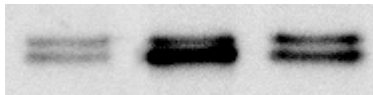

GAPDH

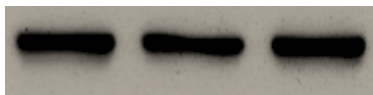

6) Caspase 3

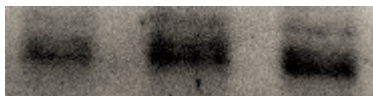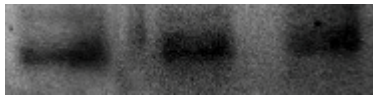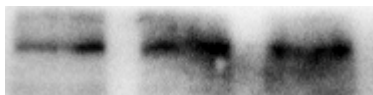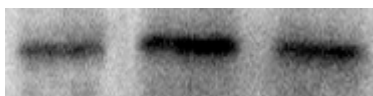

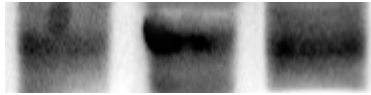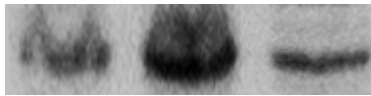

GAPDH

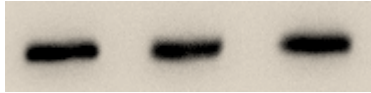

7)Lc3B

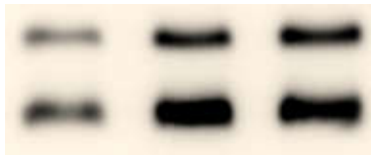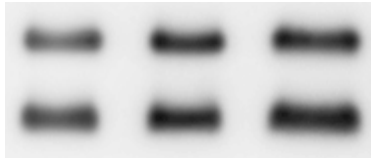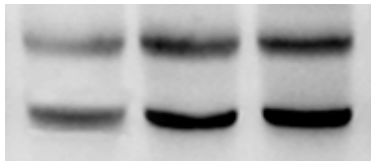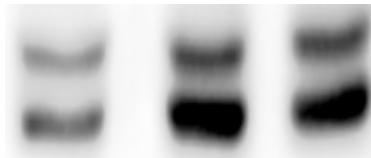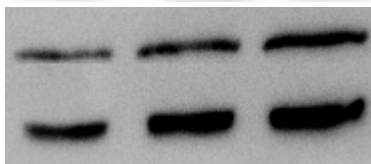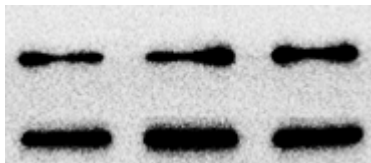

GAPDH

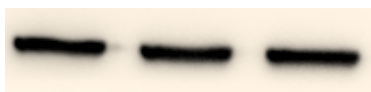

Supplement: Supplementary Materials — Western blotting after OGD/R and treatment with ECG. HBMVECs were harvested, and cell extracts were prepared. Briefly, cells were lysed in lysis buffer (20 mM Tris-pH 7.5, 150 mM NaCl, and 1% Triton X-100) followed by centrifugation for 3 min at 10,000 × g at 4°C. The supernatant was collected and transferred electrophoretically to a polyvinylidene fluoride membrane (PVDF) (Millipore, Shanghai, China). The membranes were blocked by 5% dry milk and subsequently incubated with primary antibodies against GAPDH (Abcam, UK), VEGF (Abcam, UK), Bcl-2 (Abcam, UK), BAX (CST, USA), Caspase 3 (CST, USA), mTOR (CST, USA), LC3B (Abcam, UK), and Beclin-1 (CST, USA) overnight at 4°C. The membranes were subsequently washed and incubated with secondary antibodies conjugated with horseradish peroxidase. The immunoreactive bands were visualized by enhanced chemiluminescence (Thermo Scientific, Shanghai, China) and analyzed by automatic chemiluminescence image analysis system (Tanon, China). The results were normalized to GAPDH. Results on the effect of ECG on mRNA and protein expression of VEGF, Bcl-2, BAX, LC3B, Caspase 3, mTOR, and Beclin-1 mRNA and protein expression of vascular endothelial growth factor of VEGF, cell proliferation, and apoptosis associated Bcl-2, BAX, and Caspase 3, as well as LC3B, mTOR, and Beclin-1 which is related to autophagy were measured in our study. Compared with control, mRNA expression of VEGF (Figure 4(a)) and Bcl-2 (Figure 4(c)) decreased significantly (P < 0.01), whereas BAX (Figure 4(e)), mTOR (Figure 4(g)), Beclin-1 (Figure 4(i)), Caspase 3 (Figure 4(k)), and LC3B (Figure 4(m)) mRNA expression increased significantly (P < 0.01) in the OGD/R group. ECG tends to inhibit downregulation of VEGF and Bcl2 as well as upregulation of BAX, mTOR, Beclin-1, Caspase 3, and LC3B induced by OGD/R. To investigate the effect of ECG on expression of OGD/R related proteins and the mechanism involved, we also measured protein expression of VEGF, Bcl2, BAX, mTOR, Becl [file 7824684.f1.pdf]
